# Coping strategies among family caregivers of community-dwelling older adults in Lebanon amid the economic crisis

**DOI:** 10.1371/journal.pone.0340972

**Published:** 2026-01-23

**Authors:** Zainab Barakat, Hala Sacre, Sarah El Khatib, Linda Abou Abbas, Marc Barakat, Pascale Salameh, Samar Rachidi

**Affiliations:** 1 Clinical and Epidemiological Research Laboratory, Doctoral School of Science and Technology, Lebanese University, Beirut, Lebanon; 2 Faculty of Medical Sciences, Lebanese University, Hadat, Lebanon; 3 Institut National de Santé Publique d’Épidémiologie Clinique et de Toxicologie-Liban (INSPECT-LB), Beirut, Lebanon; 4 Department of Psychiatry, American University of Beirut Medical Center, Beirut, Lebanon; 5 Faculty of Pharmacy, Lebanese University, Hadat, Lebanon; 6 Gilbert and Rose-Marie Chagoury School of Medicine, Lebanese American University, Beirut, Lebanon; 7 Department of Primary Care and Population Health, University of Nicosia Medical School, Nicosia, Cyprus; University of Sao Paulo, BRAZIL

## Abstract

**Background:**

Adopting effective and adaptable coping strategies can help reduce the distress associated with the caregiving burden. Studies assessing coping strategies among caregivers of older adults in the Arab world remain limited. This study aims to assess the coping strategies employed by caregivers of older adults in Lebanon, investigate factors associated with these coping mechanisms, and examine whether social support mediates the relationship between psychological distress and coping amid Lebanon’s ongoing economic crisis.

**Methods:**

In this cross-sectional study, 544 caregivers of community-dwelling older adults participated. Data were collected online via Google Forms and included several instruments: a socio-demographic questionnaire, the Brief Coping Orientation to Problems Experienced Inventory Scale, the Katz Index of Activities of Daily Living, the Autonomy in Daily Functioning-Contemporary Scale, the Depression, Anxiety, and Stress Scale, and the Multidimensional Scale of Perceived Social Support. Generalized linear models were used to identify factors associated with different coping strategies.

**Results:**

Caregivers primarily relied on problem-focused coping strategies to manage stress, with an average score of 5.73 (standard deviation [SD] = 1.22). Among the various coping methods, religious coping was the most frequently used, with a mean score of 6.82 (SD = 1.50), while substance use was the least commonly adopted, scoring an average of 2.57 (SD = 1.24). Higher household income was significantly associated with greater use of problem-focused coping (B = 0.352; p-value = 0.003). Additionally, higher educational attainment was linked to increased use of problem-focused (B = 0.240; p-value = 0.044) and emotion-focused coping strategies (B = 0.284; p-value = 0.024). Psychological distress was inversely related to emotion-focused and problem-focused coping but showed a positive correlation with avoidance coping. Social support was positively correlated with both emotion-focused coping (B = 0.881; p-value < 0.001) and problem-focused coping (B = 1.005; p-value < 0.001) and partially mediated the association between coping methods and psychological distress.

**Conclusion:**

Lebanese caregivers primarily rely on adaptive coping strategies to manage ongoing difficulties and rarely resort to maladaptive approaches. The findings emphasize the need for caregiver support initiatives that are culturally relevant and psychologically supportive, acknowledging the significant influence of socioeconomic status, psychological distress, and social support on coping behaviors.

## Introduction

Caring for older adults with chronic illnesses or disabilities who cannot live independently and require assistance with daily activities can adversely impact family caregivers’ physical and psychological well-being [1]. These caregivers frequently experience high levels of burden, psychological distress, and increased physical and mental health problems [2–4]. The negative health outcomes are primarily driven by the demanding nature of caregiving, compounded by the care recipients’ physical and cognitive impairments as well as behavioral disorders [5].

The implementation of effective and adaptable coping strategies serves as a protective measure to alleviate the distress experienced by caregivers due to the burden of caring [6,7]. Coping involves adjusting to challenging circumstances by utilizing cognitive and behavioral resources to address specific internal or external demands that exceed an individual’s typical capacity [8]. These strategies are highly individualized, varying according to personal experiences, available resources, preferences, motivation, and skills [9]. Despite some debate on coping classifications [10], it is generally accepted that coping falls into three primary types: problem-focused, emotion-focused, and avoidance coping strategies [11,12].

The Stress Process Model suggests that a variety of factors influence how caregivers cope [13]. These factors can be grouped into three domains: stressors, individual caregiver resources, and social resources [14]. Caregivers face many stressors that increase caregiving stress, including the deteriorated health, functional impairments, and behavioral problems of older adults [15–17]. Consequently, caregivers employ various coping strategies to manage stress, with their coping abilities influenced by demographic characteristics, relationships with care recipients, health conditions, caregiving history, and social support [14].

Most existing studies on coping strategies among caregivers of older adults tend to focus on specific subgroups, such as those caring for individuals with dementia [18–24]. Only a limited number have examined coping strategies and their associated factors in caregivers of older adults more broadly [14,25]. For instance, one study in Nigeria explored burden and coping among spouses, adult children, and adult-children-in-law caring for older adults with chronic illnesses but did not consider caregivers’ social resources. Another study in the United States investigated coping patterns among spouses and adult children of frail older adults but excluded other relatives. Furthermore, this study did not utilize an internationally validated coping assessment scale, resulting in the omission of key emotion-focused strategies such as acceptance and humor, as well as essential problem-focused strategies like positive reframing. Additionally, it included very few avoidance coping methods, neglecting critical approaches such as denial, self-distraction, and self-blame. A separate study from Spain identified the most frequently used coping strategies among caregivers of dependent older adults but did not examine predictors of these coping methods [26].

Despite the rapidly growing elderly population in Lebanon and the broader Arab region, research on the coping strategies used by family caregivers of older adults remains scarce. Currently, about 10% of Lebanon’s population is aged 65 and above, a figure projected to rise to over 27% by 2050 [27,28]. This demographic shift is unfolding amid an acute economic crisis and political instability that have severely undermined the country’s financial and healthcare systems [29]. Due to strong cultural norms emphasizing family responsibility and interdependence, Lebanese older adults primarily rely on family caregivers for social, financial, and healthcare support. However, the ongoing economic crisis—characterized by high inflation, rising public debt, and significant currency devaluation—has devastated healthcare infrastructure, reduced public health coverage, and increased out-of-pocket costs [30]. The crisis has intensified the caregiving burden, as many families cannot access formal support services. Consequently, Lebanese caregivers face the immense challenge of supporting aging relatives amid severe economic hardship.

Previous research examining coping strategies in resource-limited and crisis-affected settings has shown that adaptive strategies such as religion and problem-solving effectively alleviate caregiver stress [31–34], while maladaptive coping is linked to increased distress [32]. Our prior study on caregiver burden amid Lebanon’s economic crisis similarly identified maladaptive coping as a significant contributor to heightened burden [35]. However, comprehensive studies that thoroughly assess the types of coping strategies and their associated factors among informal caregivers in crisis contexts, particularly within developing countries, remain scarce.

Given cultural variations in coping methods [36], Lebanon’s rapidly aging population, and ongoing economic instability, investigating Lebanese caregivers’ management of growing responsibilities and influencing factors is essential. This study examines coping strategies employed by family caregivers of community-dwelling older adults in Lebanon amid the economic crisis. It also assesses relationships between caregivers’ coping strategies and socio-demographic factors, care recipient characteristics, social support, and psychological distress. Additionally, it tests whether social support mediates the link between psychological distress and coping.

Understanding these coping mechanisms will provide valuable insights into how caregivers manage caregiving challenges amid crisis conditions, informing the design of targeted interventions to reduce caregiver burden and improve psychological well-being. Expanding research to include a broader caregiver population will yield a more comprehensive understanding of their challenges and support the development of effective caregiver programs.

## Methodology

### Study design and participants

A quantitative cross-sectional study targeting caregivers of older adults was carried out in Lebanon between May and June 2024. To qualify for inclusion, participants had to be family caregivers aged 18 years or older, providing unpaid care to community-dwelling older adults aged 65 years or above residing in Lebanon. Eligible caregivers included spouses, sons, daughters, grandchildren, or other relatives who possessed basic literacy skills. Exclusion criteria comprised paid formal caregivers, family caregivers living outside Lebanon, individuals under 18 years of age, those caring for older adults outside community settings, and individuals who did not provide electronic informed consent indicating voluntary participation.

### Data collection

Data was collected using a snowball technique, and an online survey was distributed via various social media platforms (WhatsApp, Facebook, and Instagram). A brief description outlining the study’s objective, eligibility criteria, and informed consent preceded the survey. Caregivers provided demographic data and additional information via an online Google Form with the built-in “Limit to one response” feature enabled to prevent duplicate submissions. The questionnaire, provided in Arabic, Lebanon’s primary language, encompassed three sections and took approximately 20 minutes to complete. The first section collected demographic data and other relevant characteristics about the caregivers. The second section asked caregivers to report characteristics of the care recipients. The third section included scales assessing the care recipients’ functional abilities, as well as instruments evaluating caregivers’ adopted coping strategies, psychological distress, and perceived social support. The questionnaire was piloted with 15 caregivers, who reported no difficulties in understanding or comprehending the items. Consequently, no further modifications were needed.

### Sample size calculation

Using G-Power software (version 3.0.10), the minimum sample size was calculated. As the primary dependent variable is continuous, the sample size calculation was performed on the basis of a regression model. The effect size calculated was 0.0526, with an anticipated squared multiple correlation of 0.05 (R2 deviation from 0) for the omnibus test of multiple regression. The required sample size was n = 415, assuming an alpha error of 5%, 80% power, and 20 predictors in the model.

### Measures

#### Dependent variable: Coping strategies.

Caregivers’ coping strategies were assessed using the Brief Coping Orientation to Problems Experienced Inventory Scale (Brief COPE-28), which is a 28-item scale. The items are categorized into 14 coping strategies, with two each. Each item is rated on a 4-point Likert scale ranging from 1 (“I haven’t been doing it at all”) to 4 (“I have been doing it a lot”) [37]. These coping strategies are further grouped into three coping subscales: problem-focused coping (active coping, planning, instrumental support, and positive reframing); emotion-focused coping (emotional support, acceptance, religion, and humor); and avoidance coping (self-distraction, venting, denial, substance use, behavioral disengagement, and self-blame) [38–41]. Subscale scores are determined by adding up the items corresponding to each subscale. Higher subscale scores indicate the respondents’ increased propensity to employ a particular coping strategy [37]. We used the validated Arabic version of the COPE-28 scale [42], which demonstrated satisfactory internal consistency in our study. The overall Cronbach’s alpha was 0.833, with subscale values of 0.811 for problem-focused coping, 0.715 for emotion-focused coping, and 0.722 for avoidance coping.

#### Independent variables.

Data on caregiver socio-demographics included age, gender, marital status, educational level, place of residence, region (urban/rural), employment status, healthcare professional status, monthly household income, relationship to the care recipient (e.g., son/daughter, spouse, son/daughter-in-law, siblings, etc.), and living arrangement (cohabitating with or separately from the care recipient). Caregiving-related variables encompassed the daily number of hours spent providing care, the overall duration of caregiving in months, and the caregiver’s intention to continue in their role.

Care recipient variables included demographic information (age, gender, and financial status) and clinical characteristics. The clinical data covered diagnoses such as dementia and various chronic diseases, including hypertension, diabetes, cardiovascular diseases (CVDs), stroke, and transient ischemic attack. Additional conditions comprised chronic respiratory diseases (e.g., asthma, chronic bronchitis, chronic obstructive pulmonary disease), heart failure (including valve problems or replacements), chronic kidney disease, chronic liver disease (e.g., hepatitis), chronic urinary problems, cancer, arthritis, Parkinson’s disease, bedsores, chronic stomach problems (e.g., gastric ulcers), and chronic musculoskeletal conditions associated with pain or physical limitations. Based on this list, the total number of chronic diseases was recorded. In this study, multiple chronic diseases were defined as the presence of two or more chronic diseases.

Older adults’ functional independence in basic daily self-care activities (feeding, toileting, hygiene, dressing, sphincter continence, and transferring) was evaluated using the Katz Index of Activities of Daily Living (ADL), which consists of six items [43]. The Arabic version of the ADL scale has been validated among Lebanese older adults residing in nursing homes [44]. Responses were rated as 0, 0.5, or 1, with 0.5 indicating partial independence. Higher scores reflect greater functional independence levels in performing these basic self-care tasks.

Moreover, the care recipients’ ability to independently perform instrumental activities of daily living within the modern community was assessed using the 11-item Autonomy in Daily Functioning-Contemporary Scale (ADF-CS). This scale evaluates tasks such as using landlines and mobile phones, preparing food, housekeeping, shopping, traveling beyond walking distance, traveling alone abroad, managing medication and finances, using television, and operating household electrical devices. Scores on this scale range from 1 (dependent) to 2 (partially dependent) to 3 (independent), with the total score obtained by adding up all individual items. A higher total score indicates a higher level of functional independence in IADL performance. We have developed, cross-culturally adapted, and validated this scale in Lebanon [45].

Caregivers’ overall psychological distress was measured using the Depression, Anxiety, and Stress Scale–8 Items (DASS-8), a shorter form of the DASS-21. Responses to the items are rated on a 4-point scale ranging from 0 (did not apply to me at all) to 3 (applied to me very much or most of the time). The DASS-8 overall score ranges from 0 to 24, with higher scores indicating greater levels of psychological distress [46]. We used the validated Arabic version of this scale [46], demonstrating a satisfactory internal consistency in our study, with a Cronbach’s alpha of 0.908.

Social support perceived by caregivers from family, friends, and significant others was assessed using a 12-item concise research instrument called the Multidimensional Scale of Perceived Social Support (MDSPSS) [47]. Each statement is scored on a seven-point Likert scale, with responses ranging from 1 (very strongly disagree) to 7 (very strongly agree). The overall score ranged between 12 and 84 and was further categorized into three levels: low support (12–47), moderate support (48–68), and high support (69–84) [48,49]. The Arabic version, previously validated in Lebanon, was used in this study [50].

All scales included in the study were approved for use and were provided in Arabic, Lebanon’s native language.

### Data management and analysis

Data entry and analysis were performed via the statistical software SPSS, version 27.0. For descriptive analysis, means and standard deviations (SD) were reported for continuous variables with a normal distribution, whereas medians and interquartile ranges (IQR) were reported for those with a non-normal distribution. Categorical variables were presented as frequencies (n) with percentages (%). For the 14 coping strategy domains, both the mean (SD) and median (IQR) values were reported to facilitate comparison. Due to the exploratory nature of the study and sample size constraints, confirmatory factor analysis (CFA) was not conducted; however, internal consistency was evaluated using Cronbach’s alpha, with values above 0.70 indicating acceptable reliability. The three groups of coping strategies—problem-focused coping, emotion-focused coping, and avoidance coping —were the dependent variables. After normality was assessed via the Kolmogorov-Smirnov tests and graphical methods, bivariate analysis was conducted separately to investigate the associations between each dependent variable and the independent variables. For variables with a non-normal distribution, the Kruskal-Wallis H, Spearman correlation, and Mann-Whitney U tests were performed, whereas for normally distributed variables meeting the assumptions, one-way ANOVA, Pearson correlation, and independent-samples T-tests were executed. Generalized linear models (GzLM) were used to investigate the potential factors associated with the three coping strategy groups. In the linear analysis models, we included all variables showing a p-value of less than 0.2 in the bivariate analysis. Unstandardized regression coefficients (B) and their corresponding 95% confidence intervals (95% CI) were reported. Moreover, we used Model 4 in PROCESS version 5.0 to evaluate the mediating role of social support between coping methods and psychological distress. To test the statistical significance of the regression coefficient, a bootstrap procedure with 5000 samples and a 95% CI was performed. Effects were considered significant when the confidence interval did not include zero. The threshold for significance was set at a two-sided p-value of less than 0.05.

### Ethical consideration

The research followed the ethical guidelines defined in the Declaration of Helsinki [51]. All caregivers provided their online informed consent to confirm their voluntary participation. Additionally, they had the right to decline participation at any time, and their anonymity and confidentiality were rigorously protected throughout the study. Approval for the study was granted by the Institutional Review Board of the National Institute of Public Health, Clinical Epidemiology, and Toxicology—Lebanon (INSPECT-LB) with reference number 2024REC-001-INSPECT-01–13.

## Results

### Characteristics of the study participants

Our sample included 544 caregivers of community-dwelling older adults; the median age was 40 years (IQR = 15), with 84% females and 16% males. Most participants were married (66.9%), had a higher level of education (44.7%), and lived in urban areas (61.2%), especially in Beirut (30%). Among the total sample, 55.3% were employed, 16.7% were health care professionals, and 44.3% had a monthly household income of less than 250 USD.

The median level of psychological distress experienced by caregivers was 8 (IQR = 9). Approximately half of the respondents (52.2%) reported receiving moderate social support, 38.4% reported experiencing high social support, and 9.4% reported receiving low social support. The caregivers’ most common coping strategy was problem-focused (median = 5.75, IQR = 2.00; mean = 5.73, SD = 1.22), followed by emotion-focused (median = 5.50, IQR = 1.50; mean = 5.45, SD = 1.12) and avoidance coping (median = 4.17, IQR = 1.33; mean = 4.18; SD = 0.98) (Table 1).

**Table 1:** Characteristics of informal caregivers for community-dwelling older adults (N = 544).

Variables	n (%)	Median (IQR)	Mean (SD)
**Age (years)**		40.0 (15.0)	40.2 (10.6)
**Gender**			
Male	87 (16)		
Female	457 (84)		
**Marital status**			
Married	364 (66.9)		
Single	111 (20.4)		
Divorced	53 (9.7)		
Widowed	16 (2.9)		
**Educational level**			
Intermediate or lower	181 (33.3)		
Secondary	120 (22.1)		
University/postgraduate	243 (44.7)		
**Place of residence**			
Beirut	163 (30)		
Mount Lebanon	108 (19.9)		
North	43 (7.9)		
Akkar	12 (2.2)		
South	118 (21.7)		
Nabatiyeh	50 (9.2)		
Beqaa	28 (5.1)		
Baalbek-Hermel	22 (4)		
**Working status**			
Employed	301 (55.3)		
Unemployed	243 (44.7)		
**Being a healthcare professional**			
No	453 (83.3)		
Yes	91 (16.7)		
**Monthly household income**			
<250 USD	241 (44.3)		
250-500 USD	165 (30.3)		
500-1000 USD	87 (16)		
>1000 USD	51 (9.4)		
**Caregiver’s relationship with the care recipient**			
Son/daughter	337 (61.9)		
Grandchild	69 (12.7)		
Son/daughter-in-law	97 (17.8)		
Spouse	12 (2.2)		
Others	29 (5.3)		
**Perceived social support**		58.0 (17.0)	55.9 (15.4)
Low support	51 (9.4)		
Moderate support	284 (52.2)		
High support	209 (38.4)		

n: frequency, %: percentage, IQR: interquartile range, SD: standard deviation.

Most of the participants (61.9%) were sons or daughters of their care recipients, 17.8% were sons- or daughters-in-law, 12.7% were grandchildren, 2.2% were spouses, and 5.3% were others, including siblings. The median age of the 544 older adults was 75.5 years (IQR = 15). Most of them were females (70.4%), cohabitating with their caregivers (85.7%), and financially dependent on them (70%). With respect to the health and functional status of the older adults, approximately one-quarter (24.6%) had dementia, and most (88.4%) had multiple chronic diseases. The average mean (SD) number of chronic diseases was 4.08 (2.19). The median scores for ADF-CS and ADL were 18 (IQR = 8) and 4 (IQR = 3.5), respectively. Regarding caregiving characteristics, almost all caregivers (98.5%) would continue caring for older adults. The median time of daily caregiving and caregiving duration were 8 (IQR = 10) hours and 24 (IQR = 36) months, respectively. Details are presented in S1 Table.

### Types of coping strategies utilized by caregivers

Table 2 presents the median and mean scores for the 14 coping methods employed by caregivers. The most commonly utilized coping strategies included religion, planning, acceptance, positive reframing, and active coping. Among these, religion emerged as the most preferred strategy, while substance use was the least utilized coping strategy.

**Table 2:** Descriptive statistics of coping strategies among caregivers of community-dwelling older adults.

Coping strategies	Median (IQR)	Mean (SD)
**Problem-focused coping**		
Active coping	6.00 (2.00)	5.99 (1.52)
Instrumental support	4.00 (3.00)	4.62 (1.80)
Planning	6.00 (3.00)	6.30 (1.48)
Positive reframing	6.00 (3.00)	6.00 (1.67)
**Emotion-focused coping**		
Emotional support	5.00 (2.00)	4.82 (1.75)
Acceptance	6.00 (2.00)	6.02 (1.57)
Religion	8.00 (2.00)	6.82 (1.50)
Humor	4.00 (3.00)	4.14 (1.87)
**Avoidance coping**		
Self-distraction	5.00 (3.00)	5.40 (1.66)
Venting	5.00 (2.00)	4.99 (1.74)
Denial	4.00 (3.00)	3.84 (1.79)
Behavioral disengagement	4.00 (3.00)	3.79 (1.62)
Self-blame	4.00 (3.00)	4.48 (1.90)
Substance use	2.00 (0.00)	2.57 (1.24)

SD: standard deviation, IQR: interquartile range.

### Factors associated with coping strategies: Bivariate analysis

**Fig 1:**
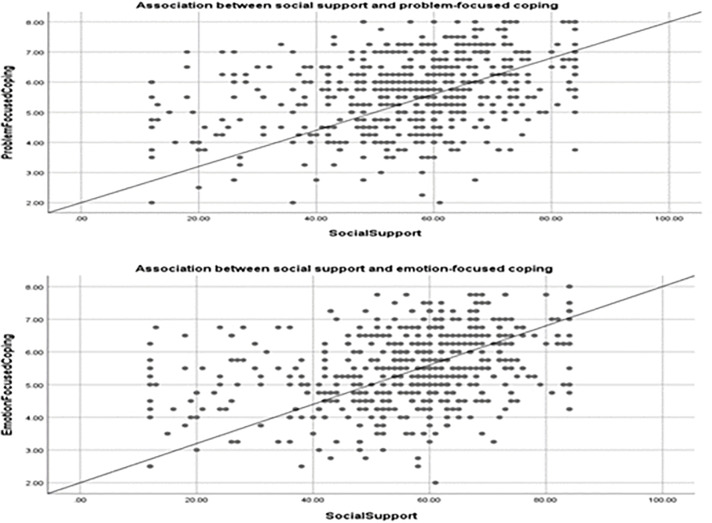
Associations of social support with problem-focused and emotion-focused coping.

S2 and S3 Tables present the results of bivariate analysis examining caregivers' three major coping strategies: problem-focused, emotion-focused, and avoidance coping. Higher educational level and monthly family income were significantly associated with greater use of problem-focused and emotion-focused coping. Psychological distress was negatively correlated with problem-focused and emotion-focused coping but positively associated with avoidance coping. Social support showed significant positive correlations with both problem-focused and emotion-focused coping (see Fig 1).

Caregivers of individuals diagnosed with dementia exhibited significantly higher scores in problem-focused coping, indicating a greater reliance on these strategies than caregivers of non-dementia care recipients. The number of chronic diseases in care recipients was positively associated with the use of avoidance coping. Additionally, avoidance coping was uniquely related to caregiver gender and their relationship to the care recipient, with female caregivers reporting significantly greater use of avoidance strategies than males.

No statistically significant associations were found between any of the three coping strategies and factors such as marital status, place of residence, region, employment status, healthcare professional status, cohabitation with care recipients, care recipients’ age and gender, caregiving characteristics (including daily hours, duration, and willingness to continue caregiving), or the financial and functional status of care recipients.

Variables with p-values below 0.2 for any coping strategy are detailed in S2 Table, while those with higher p-values are summarized in S3 Table.

### Factors associated with coping strategies: Multivariable analysis

The GzLM was conducted separately for each coping strategy dimension. Significant differences emerged in the use of problem-focused coping strategies, with caregivers holding higher education levels (university/postgraduate) reporting greater use compared to those with intermediate or lower education. Similarly, caregivers with a monthly household income between 250 and 500 USD used more problem-focused coping strategies than those earning less than 250 USD. Problem-focused coping was negatively associated with psychological distress but positively associated with the number of chronic illnesses affecting older adults. Additionally, caregivers who perceived either high or moderate levels of social support exhibited significantly greater use of problem-focused coping strategies (S4 Table).

The findings for the emotion-focused coping strategy revealed that significant predictors included educational level, social support, and psychological distress. Caregivers with higher education, greater perceived social support, and lower psychological distress reported higher scores on emotion-focused coping compared to their counterparts (S5 Table).

The results indicated that caregivers experiencing higher psychological distress were significantly more likely to use avoidance coping strategies compared to those with lower distress levels. In contrast, caregivers residing in the North/Akkar districts of Lebanon reported notably lower avoidance coping scores compared to their counterparts in the Beirut district (S6 Table).

### Mediation analyses

Mediation analyses were conducted to examine whether social support mediated the relationship between psychological distress and coping strategies (Table 3).

**Table 3:** Indirect effects of psychological distress on coping strategies through the mediating role of social support.

	Effect	Boot SE	95% CI	
			Lower	Upper
Psychological distress → social support → problem-focused coping	−0.0168	0.0036	−0.0242	−0.0102
Psychological distress → social support → emotion-focused coping	−0.0162	0.0033	−0.0229	−0.0101
Psychological distress → social support → avoidance coping	−0.0051	0.0022	−0.0099	−0.0012

Problem-focused coping: Psychological distress was negatively associated with social support (β = −0.6684, p-value < 0.001). Social support was positively associated with problem-focused coping (β = 0.0252, p-value < 0.001). The direct effect of psychological distress on problem-focused coping was negative and significant (β = −0.0195, p-value = 0.022). The indirect effect through social support was significant.

Emotion-focused coping: Psychological distress was negatively associated with social support (β = −0.6684, p-value < 0.001). Social support was positively associated with emotion-focused coping (β = 0.0243, p-value < 0.001). The direct effect of psychological distress on emotion-focused coping was negative and marginally significant (β = −0.0154, p-value = 0.0498). The indirect effect through social support was significant.

Avoidance coping: Psychological distress was negatively associated with social support (β = −0.6684, p-value < 0.001). Controlling for psychological distress, social support was positively associated with avoidance coping (β = 0.0077, p-value = 0.003). The direct effect of psychological distress on avoidance coping was positive and significant (β = 0.0703, p-value < 0.001). The indirect effect through social support was minimal but significant.

## Discussion

The burden experienced by family caregivers of older adults in Lebanon has been significantly heightened by the ongoing economic crisis, with many enduring profound financial hardship [35]. In the present study, we specifically examined the coping strategies employed by these caregivers to manage the severe economic challenges they face. Due to variations in assessment scales and conceptual differences regarding coping, comparing findings across studies remains challenging [40]. Nonetheless, our findings showed that caregivers of community-dwelling older adults predominantly preferred problem-focused coping strategies over emotion-focused ones, consistent with studies involving caregivers of individuals with dementia, Alzheimer’s disease, and schizophrenia [52–56], which have reported problem-focused coping as a primary adaptive approach for managing stress. In the Lebanese context, caregivers of older adults may favor problem-focused coping given their daily realities, which demand practical problem-solving. Financial strain, unemployment, and insufficient governmental support, including a lack of financial aid and caregiving training, compel Lebanese caregivers to adopt immediate solution-oriented strategies to handle challenges effectively.

Although problem-focused coping is generally considered more beneficial and is associated with improved outcomes, such as reduced caregiver distress and enhanced quality of life, emotion-focused coping remains commonly utilized in many situations [25,57]. In our study, religious coping emerged as the most frequently employed method, consistent with findings from caregivers of individuals with chronic illnesses, cancer, and mental health conditions [25,40,58]. In Lebanon, religious communities offer essential support and foster a strong sense of belonging. Positive religious coping, characterized by trusting in divine control [59] and accepting circumstances as “the will of God,” is linked to higher life satisfaction and improved stress management [60], and can be considered a beneficial coping method. However, religious coping may sometimes fall short in adequately addressing the underlying stressors and their effects [9], leading to mixed evidence about its overall health impact [61,62]. In contrast, negative religious coping, such as punitive religious reappraisals and religious discontent [63], is correlated with poorer health outcomes [64].

On the other hand, substance use, classified as avoidance coping, was the least utilized coping method among caregivers. This finding aligns with other studies using the brief COPE scale on caregivers of dependent older adults [25,26], stroke patients [65], cancer patients [66], and individuals with schizophrenia [40]. In Lebanon’s religiously diverse society, where many religions prohibit or discourage drug and alcohol use, this cultural context likely contributes to substance use being the least favored coping strategy. More frequently used adaptive coping methods included planning, acceptance, positive reframing, and active coping, consistent with previous research on caregivers of dependent older adults [26] and stroke survivors [65]. A review of dementia caregivers similarly identified problem-solving and acceptance as the most effective coping strategies [67]. Clinically, these results underscore the importance of supporting caregivers in identifying and implementing practical solutions to challenges in patient care while also fostering acceptance of situations beyond their control. Overall, Lebanese caregivers of community-dwelling older adults with chronic conditions predominantly employed adaptive coping methods to manage ongoing challenges, rarely resorting to maladaptive strategies such as substance use.

When included in the GzLM, household income was significantly associated only with problem-focused coping; caregivers with higher income levels may have more resources to effectively confront problems. Additionally, both problem-focused and emotion-focused coping strategies were more commonly employed by caregivers with higher educational attainment. These findings highlight the significant role of socioeconomic factors, particularly education and income, in shaping caregivers’ coping strategy choices during ongoing crises, emphasizing the importance of considering these variables in caregiver support initiatives. Consistent with prior research among dementia caregivers [67,68], problem-focused coping was negatively correlated with psychological distress. Similarly, greater use of emotion-focused coping was linked to lower psychological distress, aligning with studies reporting significant negative associations between emotion-focused strategies and stress levels [54,69]. Given the complexity and potential bidirectionality of these relationships, longitudinal studies are recommended to further elucidate how coping styles and psychological distress interact over time in caregivers of older adults.

Moreover, our study found that caregivers perceiving higher levels of social support used more problem-focused and emotion-focused coping strategies. However, no significant association was observed between social support and avoidance coping. These findings align with the limited existing research on caregivers for older adults. For instance, a study involving caregivers of dependent older adults reported a significant positive association between increased perceived social support from family and relatives and the likelihood of using emotion-focused coping strategies [14]. Similarly, research among caregivers of individuals with schizophrenia yielded comparable results [40]. Social support is widely recognized as a protective factor that correlates with positive outcomes, enhances health, and reduces psychological distress among caregivers [57]. The notably high level of social support (90.6%) observed among Lebanese caregivers, deeply rooted in the country’s strong cultural values, may reflect an important resource that helps caregivers manage the numerous challenges they face during times of crisis.

Avoidance coping is generally considered maladaptive and is linked to poorer outcomes for caregivers. In our study, Lebanese female caregivers who traditionally bear the primary responsibility for caregiving were more likely to resort to maladaptive coping strategies than males. These multitasking women, confronted with the complex and demanding care of older adults amid an ongoing economic crisis, may feel overwhelmed and helpless, which may be associated with greater reliance on ineffective coping approaches. Additionally, caregivers experiencing higher psychological distress showed greater avoidance coping. This aligns with systematic reviews showing strong associations between maladaptive coping strategies and psychological morbidity among family caregivers [70]. Moreover, prior studies suggest a bidirectional relationship between stress and avoidance coping [71]: avoidance behaviors increase stress, while heightened stress further impairs caregivers’ ability to address problems effectively, thereby reinforcing avoidance.

Care recipients’ age, gender, functional status, relationship with caregivers, and caregiving duration did not significantly predict caregivers’ coping strategies, consistent with previous studies involving caregivers of older adults with chronic illnesses [25]. Notably, both problem-focused and avoidance coping were associated with an increased number of chronic illnesses in care recipients, reflecting the complex and multifaceted nature of coping in caregiving. This finding aligns with the dynamic nature of coping, whereby caregivers continually adjust their strategies based on evolving demands and individual circumstances [57]. Therefore, longitudinal studies with extended follow-up periods are warranted to further investigate how caregivers’ coping strategies change and navigate over time.

The mediation analyses examined how social support influences the relationship between psychological distress and coping strategies, addressing a relatively under-researched area. Across all models, higher psychological distress was linked to lower perceived social support. For adaptive coping strategies, including problem-focused and emotion-focused coping, social support was positively associated, partially buffering the adverse effects of distress through significant indirect effects. Conversely, social support showed a small but positive association with avoidance coping, differing from the pattern seen with adaptive coping and contrasting typical expectations. Possible explanations include the nature of avoidance as a maladaptive strategy—social support may provide reassurance or comfort that inadvertently enables short-term avoidance rather than promoting active problem-solving. Additionally, the effect size was minimal, suggesting limited practical impact, and sample-specific or contextual factors, including cultural influences on the perception and use of social support, may contribute to this finding. Consequently, while social support partially mediates the negative impact of psychological distress on adaptive coping, its role in avoidance coping is complex and warrants further investigation.

### Limitations and strengths

This study has several limitations that should be noted. Its cross-sectional design means we can only capture a snapshot in time, making it impossible to determine cause-and-effect relationships. Future research using longitudinal designs with follow-up periods would help address this issue. The snowball sampling method may have introduced bias, limiting how well the findings represent all caregivers. Further, using online surveys might have excluded caregivers with poor internet access or low digital literacy, common in rural and low-income areas, which affects the generalizability of results. Self-reported data may also be subject to recall errors and social desirability bias, potentially skewing the associations observed. Despite these limitations, the study benefits from a broadly representative sample of family caregivers and the use of validated scales, which help reduce information bias. Studies examining coping strategies among caregivers of older adults in the Arab world are scarce. To our knowledge, this is the first study to assess coping strategies specifically among caregivers in Lebanon amid the ongoing economic crisis, providing valuable insight into caregiving in this unique context.

### Implications for practice

This study highlights important directions for practical interventions to support caregivers of older adults in Lebanon, especially amid ongoing economic challenges. Programs should focus on strengthening caregivers’ problem-solving and emotional regulation skills, such as planning, positive reframing, active coping, and acceptance, which can alleviate psychological distress and enhance well-being. Given the link between higher education, household income, and more effective coping, public health efforts must prioritize caregivers from lower socioeconomic backgrounds by providing accessible resources and tailored educational support.

Effective implementation requires collaboration between key health and humanitarian organizations, such as the Ministry of Public Health and the Lebanese Red Cross, to establish structured training programs coupled with continuous follow-up and support. Local municipalities are well-positioned to identify and connect with caregivers in marginalized and underserved communities, ensuring broad reach and inclusivity.

Furthermore, the critical role of social support suggests the value of developing caregiver support groups and expanding community-based services that provide both practical assistance and emotional solidarity. Interventions to reduce psychological distress should be complemented by raising awareness about the risks of maladaptive coping strategies, particularly avoidance, and educating caregivers on recognizing and managing caregiving stressors effectively. Tailored programs should prioritize female caregivers, those with psychological distress, and those caring for older adults with multiple chronic illnesses, as these groups are most vulnerable to maladaptive coping. To maximize impact, these programs should be accessible, affordable, and widely available to ensure caregivers receive the support they need to manage their burden more effectively.

Translating these insights into policy, community-embedded programs like Lebanon’s Balsam palliative care model demonstrate how integrating medical and psychosocial support into home care services can build resilience and capacity among caregivers. Strengthening partnerships between government agencies and NGOs will be vital to expanding access to psychosocial resources, providing continuous support, and helping caregivers meet increasing care demands amid Lebanon’s socio-economic adversities.

This comprehensive approach can improve caregiver well-being, sustain their crucial role, and ultimately enhance care outcomes for older adults in Lebanon’s complex context.

## Conclusion

The present study sheds light on how family caregivers of community-dwelling older adults in Lebanon cope with stress amid a prolonged economic crisis. These caregivers primarily use adaptive coping strategies to manage ongoing challenges, rarely resorting to maladaptive ones. Our findings revealed the critical roles of socioeconomic status, psychological distress, and social support in shaping coping behaviors during difficult times. They also emphasize the need to design culturally tailored caregiver support programs that integrate government and humanitarian initiatives focused on establishing social support networks, psychological support services, and community-based education. By addressing a substantial gap in the Arab caregiving literature, especially in the context of financial adversity, this research enhances understanding of the caregiving challenges in this understudied setting. Future research should examine how coping strategies evolve over different stages of caregiving and undertake longitudinal studies to explore the changing relationship between coping mechanisms and psychological well-being over time.

## Supporting information

S1 Table Characteristics of study participants. (DOCX)

S2 Table Bivariate analysis: Coping strategy variables with p < 0.2. (DOCX)

S3 Table Bivariate analysis: Coping strategy variables with p ≥ 0.2. (DOCX)

S4 Table Factors associated with problem-focused coping strategies. (DOCX)

S5 Table Factors associated with emotion-focused coping strategies. (DOCX)

S6 Table Factors associated with avoidance coping strategies. (DOCX)

S1 Dataset Study dataset. (XLSX)

## References

[1] Schulz R, Sherwood PR. Physical and mental health effects of family caregiving. Am J Nurs. 2008;108(9 Suppl):23–7; quiz 27. doi: 10.1097/01.NAJ.0000336406.45248.4c 18797217 PMC2791523

[2] Vitaliano PP, Zhang J, Scanlan JM. Is caregiving hazardous to one’s physical health? A meta-analysis. Psychol Bull. 2003;129(6):946–72. doi: 10.1037/0033-2909.129.6.946 14599289

[3] Burton LC, Zdaniuk B, Schulz R, Jackson S, Hirsch C. Transitions in spousal caregiving. Gerontologist. 2003;43(2):230–41. doi: 10.1093/geront/43.2.230 12677080

[4] Or R, Kartal A. Influence of caregiver burden on well-being of family member caregivers of older adults. Psychogeriatrics. 2019;19(5):482–90. doi: 10.1111/psyg.12421 30854774

[5] Pinquart M, Sörensen S. Associations of stressors and uplifts of caregiving with caregiver burden and depressive mood: a meta-analysis. J Gerontol B Psychol Sci Soc Sci. 2003;58(2):P112-28. doi: 10.1093/geronb/58.2.p112 12646594

[6] Huang M-F, Huang W-H, Su Y-C, Hou S-Y, Chen H-M, Yeh Y-C, et al. Coping Strategy and Caregiver Burden Among Caregivers of Patients With Dementia. Am J Alzheimers Dis Other Demen. 2015;30(7):694–8. doi: 10.1177/1533317513494446 23813690 PMC10852617

[7] Iavarone A, Ziello AR, Pastore F, Fasanaro AM, Poderico C. Caregiver burden and coping strategies in caregivers of patients with Alzheimer’s disease. Neuropsychiatr Dis Treat. 2014;10:1407–13. doi: 10.2147/NDT.S58063 25114532 PMC4122550

[8] Folkman S, Lazarus RS. An analysis of coping in a middle-aged community sample. J Health Soc Behav. 1980;21(3):219–39. doi: 10.2307/2136617 7410799

[9] Ismael N, Jaber A, Malkawi S, Al Awady S, Ismael T. Exploring coping strategies among caregivers of children who have survived paediatric cancer in Jordan. BMJ Paediatrics Open. 2024;8(1). 10.1136/bmjpo-2023-002453 PMC11015291 38604770

[10] Powers DV, Gallagher-Thompson D, Kraemer HC. Coping and depression in Alzheimer’s caregivers: longitudinal evidence of stability. J Gerontol B Psychol Sci Soc Sci. 2002;57(3):P205-11. doi: 10.1093/geronb/57.3.p205 11983731

[11] Endler NS, Parker JD. Multidimensional assessment of coping: a critical evaluation. J Pers Soc Psychol. 1990;58(5):844–54. doi: 10.1037//0022-3514.58.5.844 2348372

[12] Folkman S, Lazarus RS. If it changes it must be a process: study of emotion and coping during three stages of a college examination. J Pers Soc Psychol. 1985;48(1):150–70. doi: 10.1037//0022-3514.48.1.150 2980281

[13] Pearlin LI, Mullan JT, Semple SJ, Skaff MM. Caregiving and the stress process: an overview of concepts and their measures. Gerontologist. 1990;30(5):583–94. doi: 10.1093/geront/30.5.583 2276631

[14] Lin I-F, Wu H-S. Patterns of coping among family caregivers of frail older adults. Res Aging. 2014;36(5):603–24. doi: 10.1177/0164027513513271 25651512 PMC4318268

[15] Chappell NL, Dujela C. Caregivers--who copes how? Int J Aging Hum Dev. 2009;69(3):221–44. 20041567 10.2190/AG.69.3.d

[16] Haley WE, Roth DL, Coleton MI, Ford GR, West CA, Collins RP, et al. Appraisal, coping, and social support as mediators of well-being in black and white family caregivers of patients with Alzheimer’s disease. J Consult Clin Psychol. 1996;64(1):121–9. doi: 10.1037//0022-006x.64.1.121 8907091

[17] Kramer BJ. Expanding the conceptualization of caregiver coping: the importance of relationship-focused coping strategies. Fam Relat. 1993;42(4):383. doi: 10.2307/585338

[18] Chen H-M, Huang M-F, Yeh Y-C, Huang W-H, Chen C-S. Effectiveness of coping strategies intervention on caregiver burden among caregivers of elderly patients with dementia. Psychogeriatrics. 2015;15(1):20–5. doi: 10.1111/psyg.12071 25515800

[19] Shaw WS, Patterson TL, Semple SJ, Grant I, Yu ES, Zhang M, et al. A cross-cultural validation of coping strategies and their associations with caregiving distress. Gerontologist. 1997;37(4):490–504. doi: 10.1093/geront/37.4.490 9279038

[20] Lee K, Cassidy J, Zhao J, Mitchell J. Understanding challenges and coping strategies experienced by Chinese American family caregivers of persons with dementia. J Appl Gerontol. 2023;42(5):919–27. doi: 10.1177/07334648221142600 36437784

[21] Owokuhaisa J, Kamoga R, Musinguzi P, Muwanguzi M, Natukunda S, Mubangizi V, et al. Burden of care and coping strategies among informal caregivers of people with behavioral and psychological symptoms of dementia in rural south-western Uganda. BMC Geriatr. 2023;23(1):475. doi: 10.1186/s12877-023-04129-0 37553634 PMC10408158

[22] Rote SM, Moon HE, Kacmar AM, Moore S. Exploring coping strategies and barriers in dementia care: a mixed-methods study of African American family caregivers in Kentucky. J Appl Gerontol. 2022;41(8):1851–9. doi: 10.1177/07334648221093618 35543172

[23] Yuan Q, Wang P, Tan TH, Devi F, Poremski D, Magadi H, et al. Coping patterns among primary informal dementia caregivers in singapore and its impact on caregivers-implications of a latent class analysis. Gerontologist. 2021;61(5):680–92. doi: 10.1093/geront/gnaa080 32592582 PMC8276612

[24] Balbim GM, Marques IG, Cortez C, Magallanes M, Rocha J, Marquez DX. Coping strategies utilized by middle-aged and older latino caregivers of loved ones with Alzheimer’s disease and related dementia. J Cross Cult Gerontol. 2019;34(4):355–71. doi: 10.1007/s10823-019-09390-8 31705279

[25] Faronbi JO. Correlate of burden and coping ability of caregivers of older adults with chronic illness in Nigeria. Scand J Caring Sci. 2018;32(4):1288–96. doi: 10.1111/scs.12572 29691887

[26] Pérez-Cruz M, Parra-Anguita L, López-Martínez C, Moreno-Cámara S, Del-Pino-Casado R. Coping and anxiety in caregivers of dependent older adult relatives. Int J Environ Res Public Health. 2019;16(9):1651. doi: 10.3390/ijerph16091651 31083624 PMC6539635

[27] Hussein S, Ismail M. Ageing and elderly care in the Arab region: policy challenges and opportunities. Ageing Int. 2017;42(3):274–89. doi: 10.1007/s12126-016-9244-8 28890585 PMC5569126

[28] UNFPA-Lebanon. The rights and wellbeing of older persons in Lebanon; 2022 [cited 2025 May 10]. Available from: https://lebanon.unfpa.org/en/publications/rights-and-wellbeing-older-persons-lebanon

[29] Cherfane M, Boueri M, Issa E, Abdallah R, Hamam A, Sbeity K, et al. Unveiling the unseen toll: exploring the impact of the Lebanese economic crisis on the health-seeking behaviors in a sample of patients with diabetes and hypertension. BMC Public Health. 2024;24(1):628. doi: 10.1186/s12889-024-18116-6 38413883 PMC10900622

[30] Devi S. Economic crisis hits Lebanese health care. Lancet. 2020;395(10224):548. doi: 10.1016/S0140-6736(20)30407-4 32087781

[31] Daliri DB, Aninanya GA, Laari TT, Abagye N, Afaya A. Coping strategies used by informal family caregivers of individuals living with mental illness in the Upper East Region of Ghana: a qualitative study. BMJ Open. 2024;14(7):e084791. doi: 10.1136/bmjopen-2024-084791 39079917 PMC11293397

[32] Oleas Rodríguez DA, Yong Peña C, Garza Olivares X, Teixeira Filho FS, Lucero Córdova JE, Salas Naranjo AJ. Emotional coping strategies for informal caregivers of hospitalized patients: a study of distress and overload. Psychol Res Behav Manag. 2024;17:725–34. 38410381 10.2147/PRBM.S443200 PMC10895987

[33] Abudayya A. Voices from Gaza: coping strategies during the war on the Gaza Strip- a qualitative study. Ment Health Prev. 2025;39:200443. doi: 10.1016/j.mhp.2025.200443

[34] Hebert RS, Dang Q, Schulz R. Religious beliefs and practices are associated with better mental health in family caregivers of patients with dementia: findings from the REACH study. Am J Geriatr Psychiatry. 2007;15(4):292–300. doi: 10.1097/01.JGP.0000247160.11769.ab 17158632

[35] Barakat Z, Sacre H, Khatib S, Hajj A, Bou Malham C, Haddad C, et al. Examining burden among caregivers of community-dwelling older adults in Lebanon. Sci Rep. 2025;15(1):22775. doi: 10.1038/s41598-025-05626-5 40594243 PMC12218568

[36] Kuo BCH. Culture’s consequences on coping: theories, evidences, and dimensionalities. JCCP. 2011;42(6):1084–100.

[37] Carver CS. You want to measure coping but your protocol’s too long: consider the brief COPE. Int J Behav Med. 1997;4(1):92–100. doi: 10.1207/s15327558ijbm0401_6 16250744

[38] Carver CS, Scheier MF, Weintraub JK. Assessing coping strategies: a theoretically based approach. J Pers Soc Psychol. 1989;56(2):267–83. doi: 10.1037//0022-3514.56.2.267 2926629

[39] Cooper C, Katona C, Livingston G. Validity and reliability of the brief COPE in carers of people with dementia: the LASER-AD study. J Nerv Ment Dis. 2008;196(11):838–43. doi: 10.1097/NMD.0b013e31818b504c 19008735

[40] Kamarulbahri TMST, Ariaratnam S, Nikmat AW, Abdullah NN, Khing TL. Coping strategies and their associated factors among caregivers of patients with schizophrenia in Kuantan, Malaysia. Front Psychiatry. 2022;13:1004034. doi: 10.3389/fpsyt.2022.1004034 36245870 PMC9556896

[41] Welbourne JL, Eggerth D, Hartley TA, Andrew ME, Sanchez F. Coping strategies in the workplace: relationships with attributional style and job satisfaction. J Vocat Behav. 2007;70(2):312–25. doi: 10.1016/j.jvb.2006.10.006

[42] Alghamdi M. Cross-cultural validation and psychometric properties of the Arabic Brief COPE in Saudi population. Med J Malaysia. 2020;75(5):502–9. 32918417

[43] Katz S, Ford AB, Moskowitz RW, Jackson BA, Jaffe MW. Studies of illness in the aged. The index of ADL: a standardized measure of biological and psychosocial function. JAMA. 1963;185:914–9. 14044222 10.1001/jama.1963.03060120024016

[44] Nasser R, Doumit J. Validity and reliability of the Arabic version of activities of daily living (ADL). BMC Geriatr. 2009;9:11. doi: 10.1186/1471-2318-9-11 19327172 PMC2670307

[45] Barakat Z, Sacre H, Khatib S, Hajj A, BouMalham C, Haddad C, et al. A contemporary tool for assessing instrumental activities of daily living: validation of a caregiver-reported scale for non-institutionalized older adults. PLoS One. 2025;20(5):e0322554. doi: 10.1371/journal.pone.0322554 40333802 PMC12057986

[46] Ali AM, Hori H, Kim Y, Kunugi H. The depression anxiety stress scale 8-items expresses robust psychometric properties as an ideal shorter version of the depression anxiety stress scale 21 among healthy respondents from three continents. Front Psychol. 2022;13. 10.3389/fpsyg.2022.799769 PMC9044488 35496141

[47] Zimet GD, Powell SS, Farley GK, Werkman S, Berkoff KA. Psychometric characteristics of the multidimensional scale of perceived social support. J Pers Assess. 1990;55(3–4):610–7. doi: 10.1080/00223891.1990.9674095 2280326

[48] Grey I, Arora T, Thomas J, Saneh A, Tohme P, Abi-Habib R. The role of perceived social support on depression and sleep during the COVID-19 pandemic. Psychiatry Res. 2020;293:113452. doi: 10.1016/j.psychres.2020.113452 32977047 PMC7500407

[49] Han Y, Hu D, Liu Y, Caihong Lu, Luo Z, Zhao J, et al. Coping styles and social support among depressed Chinese family caregivers of patients with esophageal cancer. Eur J Oncol Nurs. 2014;18(6):571–7. doi: 10.1016/j.ejon.2014.07.002 25263069

[50] Fekih-Romdhane F, Fawaz M, Hallit R, Sawma T, Obeid S, Hallit S. Psychometric properties of an Arabic translation of the multidimensional social support scale (MSPSS) in a community sample of adults. BMC Psychiatry. 2023;23(1):432. doi: 10.1186/s12888-023-04937-z 37316897 PMC10265861

[51] Williams JR. The declaration of Helsinki and public health. Bull World Health Organ. 2008;86(8):650–2. doi: 10.2471/blt.08.050955 18797627 PMC2649471

[52] Snyder CM, Fauth E, Wanzek J, Piercy KW, Norton MC, Corcoran C, et al. Dementia caregivers’ coping strategies and their relationship to health and well-being: the Cache County Study. Aging Ment Health. 2015;19(5):390–9. doi: 10.1080/13607863.2014.939610 25093439 PMC4845636

[53] Zucchella C, Bartolo M, Pasotti C, Chiapella L, Sinforiani E. Caregiver burden and coping in early-stage Alzheimer disease. Alzheimer Dis Assoc Disord. 2012;26(1):55–60. doi: 10.1097/WAD.0b013e31821aa6de 21537145

[54] Cooper C, Katona C, Orrell M, Livingston G. Coping strategies, anxiety and depression in caregivers of people with Alzheimer’s disease. Int J Geriatr Psychiatry. 2008;23(9):929–36. doi: 10.1002/gps.2007 18383189

[55] Kate N, Grover S, Kulhara P, Nehra R. Relationship of caregiver burden with coping strategies, social support, psychological morbidity, and quality of life in the caregivers of schizophrenia. Asian J Psychiatr. 2013;6(5):380–8. doi: 10.1016/j.ajp.2013.03.014 24011684

[56] Chadda RK, Singh TB, Ganguly KK. Caregiver burden and coping: a prospective study of relationship between burden and coping in caregivers of patients with schizophrenia and bipolar affective disorder. Soc Psychiatry Psychiatr Epidemiol. 2007;42(11):923–30. doi: 10.1007/s00127-007-0242-8 17700975

[57] Hawken T, Turner-Cobb J, Barnett J. Coping and adjustment in caregivers: a systematic review. Health Psychol Open. 2018;5(2):2055102918810659. doi: 10.1177/2055102918810659 30450216 PMC6236498

[58] Eze NC, Ezeugwu CG, Eze RN, Soronnadi CN, Orji CJ, Chime OH. Caregiving burden and coping strategies among informal caregivers of cancer patients in Nigeria: from duty to distress. Int J Public Health. 2025;70:1607735. doi: 10.3389/ijph.2025.1607735 40290654 PMC12021601

[59] Ogunyemi A, Umoru AK, Alabi AO, Adegboyega BC, Otokpa E. Caregiving burden among informal caregivers of cancer patients in Lagos, Nigeria. 2021.

[60] Vinci C, Reblin M, Bulls H, Malkhasyan L, Jim H, Pidala J, et al. Understanding coping strategies of cancer caregivers to inform mindfulness-based interventions: a qualitative study. Eur J Integr Med. 2019;30:100936. doi: 10.1016/j.eujim.2019.100936

[61] Haghighi F. Correlation between religious coping and depression in cancer patients. Psychiatr Danub. 2013;25(3):236–40. 24048390

[62] Ng GC, Mohamed S, Sulaiman AH, Zainal NZ. Anxiety and depression in cancer patients: the association with religiosity and religious coping. J Relig Health. 2017;56(2):575–90. doi: 10.1007/s10943-016-0267-y 27287259

[63] Hebert R, Zdaniuk B, Schulz R, Scheier M. Positive and negative religious coping and well-being in women with breast cancer. J Palliat Med. 2009;12(6):537–45. doi: 10.1089/jpm.2008.0250 19508140 PMC2789454

[64] Juengst SB, Smith ML, Wilmoth K, Wright B, Han G, Supnet C, et al. Problem-solving training to improve caregiver burden and depressive symptoms among dementia caregivers: personal and clinical factors of responders vs. non-responders. Front Public Health. 2025;13:1682373. doi: 10.3389/fpubh.2025.1682373 41142740 PMC12549259

[65] Qiu Y, Li S. Stroke: coping strategies and depression among Chinese caregivers of survivors during hospitalisation. J Clin Nurs. 2008;17(12):1563–73. doi: 10.1111/j.1365-2702.2007.02156.x 18482118

[66] Long NX, Ngoc NB, Phung TT, Linh DTD, Anh TN, Hung NV, et al. Coping strategies and social support among caregivers of patients with cancer: a cross-sectional study in Vietnam. AIMS Public Health. 2020;8(1):1–14. doi: 10.3934/publichealth.2021001 33575403 PMC7870390

[67] Kneebone II, Martin PR. Coping and caregivers of people with dementia. Br J Health Psychol. 2003;8(Pt 1):1–17. doi: 10.1348/135910703762879174 12643813

[68] Di Mattei VE, Prunas A, Novella L, Marcone A, Cappa SF, Sarno L. The burden of distress in caregivers of elderly demented patients and its relationship with coping strategies. Neurol Sci. 2008;29(6):383–9. doi: 10.1007/s10072-008-1047-6 19083150

[69] Li R, Cooper C, Barber J, Rapaport P, Griffin M, Livingston G. Coping strategies as mediators of the effect of the START (strategies for RelaTives) intervention on psychological morbidity for family carers of people with dementia in a randomised controlled trial. J Affect Disord. 2014;168:298–305. doi: 10.1016/j.jad.2014.07.008 25083601

[70] Li R, Cooper C, Bradley J, Shulman A, Livingston G. Coping strategies and psychological morbidity in family carers of people with dementia: a systematic review and meta-analysis. J Affect Disord. 2012;139(1):1–11. doi: 10.1016/j.jad.2011.05.055 21723617

[71] Cooper C, Katona C, Orrell M, Livingston G. Coping strategies and anxiety in caregivers of people with Alzheimer’s disease: the LASER-AD study. J Affect Disord. 2006;90(1):15–20. doi: 10.1016/j.jad.2005.08.017 16337688

