# Supplementary material for: Coping strategies among family caregivers of community-dwelling older adults in Lebanon amid the economic crisis
Source: PLoS One. 2026 Jan 23;21(1):e0340972. doi: 10.1371/journal.pone.0340972 (PMC12829931; doi:10.1371/journal.pone.0340972)
Supplement: S1 Table — (DOCX) [file pone.0340972.s001.docx]

**S1 Table. Characteristics of the study participants**

| **Variables** | **n (%)** | **Median (IQR)** |
| --- | --- | --- |
| **Living Region Category** |  |  |
| Urban | 333 (61.2) |  |
| Rural | 211 (38.8) |  |
| **Living with the care recipient** |  |  |
| No | 80 (14.7) |  |
| Yes, sometimes | 154 (28.3) |  |
| Yes, always | 310 (57) |  |
| **Daily caregiving time (hour)** |  | 8.00 (10.00) |
| **Caregiving duration (month)** |  | 24.00 (36.00) |
| **Willingness to continue caregiving** |  |  |
| No | 8 (1.5) |  |
| Yes | 536 (98.5) |  |
| **Care recipients’ characteristics** | | |
| **Age** |  | 75.5 (15) |
| **Gender** |  |  |
| Male | 161 (29.6) |  |
| Female | 383 (70.4) |  |
| **Number of chronic diseases** |  | 4.08 (2.19) |
| **Financial status** | | |
| Independent | 168 (30.9) |  |
| Dependent on caregiver | 376 (69.1) |  |
| **Dementia** | | |
| Absent | 410 (75.4) |  |
| Present | 134 (24.6) |  |
| **Functional status** | | |
| ADF disability |  | 18.00 (8.00) |
| ADL disability |  | 4.00 (3.50) |
