# Supplementary material for: Coping strategies among family caregivers of community-dwelling older adults in Lebanon amid the economic crisis
Source: PLoS One. 2026 Jan 23;21(1):e0340972. doi: 10.1371/journal.pone.0340972 (PMC12829931; doi:10.1371/journal.pone.0340972)
Supplement: S2 Table — (DOCX) [file pone.0340972.s002.docx]

**S2 Table.** Factors associated with coping strategies among caregivers of community-dwelling older adults: bivariate analysis

| **Coping strategies** | **Problem-focused coping** | | **Emotion-focused coping** | | **Avoidance coping** | |
| --- | --- | --- | --- | --- | --- | --- |
| **Variables** | **Mean (SD)/r*** | **P-value** | **Mean (SD)/r*** | **P-value** | **Mean (SD)/r*** | **P-value** |
| **Age** | 1.00* | 0.206 | 0.005* | 0.915 | 0.080* | 0.064 |
| **Gender** |  | 0.988 |  | 0.967 |  | **0.001** |
| Male | 5.73 (1.29) |  | 5.44 (1.17) |  | 3.87 (0.93) |  |
| Female | 5.73 (1.21) |  | 5.45 (1.11) |  | 4.24 (0.98) |  |
| **Educational level** |  | **0.016** |  | **0.019** |  | 0.515 |
| Intermediate or lower | 5.54 (1.17) |  | 5.26 (1.11) |  | 4.25 (1.08) |  |
| Secondary | 5.75 (1.29) |  | 5.52 (1.16) |  | 4.21 (1.05) |  |
| University/postgraduate | 5.86 (1.21) |  | 5.55 (1.09) |  | 4.11 (0.86) |  |
| **Place of residence** |  | 0.672 |  | 0.131 |  | 0.170 |
| Beirut | 5.77 (1.27) |  | 5.51 (1.10) |  | 4.27 (1.07) |  |
| Mount Lebanon | 5.76 (1.26) |  | 5.41 (1.26) |  | 4.10 (0.95) |  |
| North | 5.49 (1.20) |  | 5.17 (1.04) |  | 4.25 (1.04) |  |
| Akkar | 5.10 (1.52) |  | 4.73 (1.09) |  | 3.42 (0.96) |  |
| South | 5.81 (1.04) |  | 5.54 (1.00) |  | 4.21 (0.92) |  |
| Nabatiyeh | 5.77 (1.24) |  | 5.55 (1.08) |  | 4.10 (0.82) |  |
| Beqaa | 5.70 (1.34) |  | 5.62 (1.21) |  | 4.11 (0.83) |  |
| Baalbek-Hermel | 5.57 (1.19) |  | 5.20 (1.20) |  | 4.26 (1.03) |  |
| **Working status** |  | 0.152 |  | 0.913 |  | 0.185 |
| Employed | 5.78 (1.27) |  | 5.44 (1.16) |  | 4.13 (0.97) |  |
| Unemployed | 5.66 (1.15) |  | 5.45 (1.07) |  | 4.24 (0.99) |  |
| **Being a healthcare professional** |  | 0.213 |  | 0.123 |  | 0.332 |
| No | 5.69 (1.22) |  | 5.42 (1.13) |  | 4.16 (0.97) |  |
| Yes | 5.90 (1.19) |  | 5.61 (1.08) |  | 4.27 (1.04) |  |
| **Monthly household income** |  | **0.003** |  | **0.020** |  | 0.354 |
| <250 USD | 5.52 (1.29) |  | 5.30 (1.19) |  | 4.17 (1.10) |  |
| 250-500 USD | 6.02 (1.05) |  | 5.62 (0.97) |  | 4.25 (0.83) |  |
| 500-1000 USD | 5.69 (1.25) |  | 5.47 (1.08) |  | 4.16 (0.97) |  |
| >1000USD | 5.80 (1.13) |  | 5.54 (1.23) |  | 4.03 (0.84) |  |
| **Caregiver’s relationship with the care recipient** |  | 0.606 |  | 0.942 |  | **0.012** |
| Grandchild | 5.82 (1.34) |  | 5.43 (1.19) |  | 4.01 (0.93) |  |
| Son/daughter | 5.68 (1.21) |  | 5.44 (1.11) |  | 4.12 (0.93) |  |
| Son/daughter-in-law | 5.72 (1.20) |  | 5.53 (1.16) |  | 4.35(1.08) |  |
| Spouse | 6.062 (0.89) |  | 5.46 (0.88) |  | 4.78 (1.12) |  |
| Others | 5.91 (1.22) |  | 5.34 (1.08) |  | 4.44 (1.08) |  |
| **Care recipient age** | 0.057* | 0.184 | 0.030* | 0.493 | -0.054* | 0.212 |
| **Daily caregiving time(hour)** | 0.027* | 0.539 | -0.049* | 0.257 | 0.058* | 0.182 |
| **Psychological distress** | -0.181* | **<0.001** | -0.177* | **<0.001** | 0.370* | **<0.001** |
| **Perceived social support** |  | **<0.001** |  | **<0.001** |  | 0.981 |
| Low support | 5.00 (1.24) |  | 4.90 (1.14) |  | 4.20 (1.05) |  |
| Moderate support | 5.54 (1.17) |  | 5.25 (1.06) |  | 4.18 (0.96) |  |
| High support | 6.16 (1.13) |  | 5.86 (1.07) |  | 4.17 (0.99) |  |
| **Number of chronic diseases** | 0.081* | 0.058 | 0.055* | 0.201 | 0.086* | **0.044** |
| **Dementia** |  | **0.028** |  | 0.224 |  | 0.301 |
| Absent | 5.67 (1.21) |  | 5.42 (1.11) |  | 4.15 (0.96) |  |
| Present | 5.90 (1.24) |  | 5.55 (1.16) |  | 4.26 (1.04) |  |
| **ADL score** | -0.032* | 0.457 | 0.006* | 0.894 | -0.063* | 0.144 |

SD: standard deviation, r*: correlation coefficient; a P-value of less than 0.05 was considered significant.
