# Supplementary material for: Coping strategies among family caregivers of community-dwelling older adults in Lebanon amid the economic crisis
Source: PLoS One. 2026 Jan 23;21(1):e0340972. doi: 10.1371/journal.pone.0340972 (PMC12829931; doi:10.1371/journal.pone.0340972)
Supplement: S3 Table — (DOCX) [file pone.0340972.s003.docx]

**S3 Table.** Factors associated with coping strategies among caregivers of community-dwelling older adults: bivariate analysis

| **Coping strategies** | **Problem-focused coping** | | **Emotion-focused coping** | | **Avoidance coping** | |
| --- | --- | --- | --- | --- | --- | --- |
| **Variables** | **Mean (SD)/r*** | **P-value** | **Mean (SD)/r*** | **P-value** | **Mean (SD)/r*** | **P-value** |
| **Marital status** |  | 0.710 |  | 0.795 |  | 0.680 |
| Married | 5.74 (1.21) |  | 5.48 (1.10) |  | 4.15 (1.00) |  |
| Single | 5.77 (1.26) |  | 5.42 (1.24) |  | 4.20 (0.93) |  |
| Divorced | 5.54 (1.24) |  | 5.31 (1.02) |  | 4.25 (0.85) |  |
| Widowed | 5.69 (0.10) |  | 5.47 (1.04) |  | 4.42 (1.24) |  |
| **Living Region Category** |  | 0.231 |  | 0.735 |  | 0.243 |
| Urban | 5.77 (1.24) |  | 5.44 (1.12) |  | 4.22 (1.01) |  |
| Rural | 5.66 (1.18) |  | 5.47 (1.12) |  | 4.12 (0.93) |  |
| **Living with the care recipient** |  | 0.623 |  | 0.263 |  | 0.755 |
| No | 5.85 (1.24) |  | 5.63 (1.05) |  | 4.12 (0.90) |  |
| Yes, sometimes | 5.73 (1.16) |  | 5.45(1.06) |  | 4.16 (1.01) |  |
| Yes, always | 5.70 (1.24) |  | 5.40 (1.16) |  | 4.20 (0.99) |  |
| **Care recipients’ gender** |  | 0.624 |  | 0.846 |  | 0.371 |
| Male | 5.68 (1.30) |  | 5.46 (1.17) |  | 4.24 (1.03) |  |
| Female | 5.75 (1.18) |  | 5.44 (1.10) |  | 4.15 (0.96) |  |
| **Caregiving duration (month)** | 0.035* | 0.415 | 0.018 | 0.685 | -0.054 | 0.216 |
| **Willingness to continue caregiving** |  | 0.423 |  | 0.775 |  | 0.454 |
| No | 6.06 (1.32) |  | 5.56 (1.41) |  | 4.44 (1.26) |  |
| Yes | 5.72 (1.22) |  | 5.45 (1.12) |  | 4.18 (0.97) |  |
| **Financial status** |  | 0.925 |  | 0.890 |  | 0.745 |
| Independent | 5.70 (1.27) |  | 5.46 (1.14) |  | 4.16 (0.95) |  |
| Dependent on caregiver | 5.74 (1.19) |  | 5.44 (1.11) |  | 4.19 (0.99) |  |
| ADF disability | 0.017* | 0.697 | 0.009 | 0.832 | -0.025 | 0.563 |

SD: standard deviation, r*: correlation coefficient; a P-value of less than 0.05 was considered significant.
