# Supplementary material for: Coping strategies among family caregivers of community-dwelling older adults in Lebanon amid the economic crisis
Source: PLoS One. 2026 Jan 23;21(1):e0340972. doi: 10.1371/journal.pone.0340972 (PMC12829931; doi:10.1371/journal.pone.0340972)
Supplement: S4 Table — (DOCX) [file pone.0340972.s004.docx]

**S4 Table.** Factors associated with problem-focused coping strategies among caregivers of community-dwelling older adults

|  | **Unstandardized Coefficients B** | **P-value** | **95.0% Confidence Interval for B** | |
| --- | --- | --- | --- | --- |
|  |  |  | **Lower Bound** | **Upper Bound** |
| Educational level (secondary vs. intermediate or lower) | 0.235 | 0.079 | -0.027 | 0.498 |
| Educational level (university/postgraduate vs. intermediate or lower) | 0.240 | **0.044** | 0.007 | 0.472 |
| Working status | -0.029 | 0.769 | -0.226 | 0.167 |
| Monthly household income (250 to 500 vs. < 250 USD) | 0.352 | **0.003** | 0.119 | 0.585 |
| Monthly household income (500 to 1000 vs. < 250 USD) | -0.014 | 0.927 | 0.281 | -0.309 |
| Monthly household income (> 1000 vs. < 250 USD) | 0.071 | 0.700 | 0.434 | -0.291 |
| Care recipients’ age | 0.000 | 0.941 | -0.011 | 0.011 |
| Psychological distress | -0.022 | **0.014** | -0.039 | -0.004 |
| Social support (moderate vs. low) | 0.457 | **0.010** | 0.109 | 0.805 |
| Social support (high vs. low) | 1.005 | **<0.001** | 0.644 | 1.366 |
| Number of chronic diseases | 0.060 | **0.007** | 0.016 | 0.104 |
| Dementia | 0.224 | 0.053 | -0.003 | 0.451 |

A P-value of less than 0.05 was considered significant.
