# Supplementary material for: Coping strategies among family caregivers of community-dwelling older adults in Lebanon amid the economic crisis
Source: PLoS One. 2026 Jan 23;21(1):e0340972. doi: 10.1371/journal.pone.0340972 (PMC12829931; doi:10.1371/journal.pone.0340972)
Supplement: S5 Table — (DOCX) [file pone.0340972.s005.docx]

**S5 Table.** Factors associated with emotion-focused coping strategies among caregivers of community-dwelling older adults

|  | **Unstandardized Coefficients B** | **P-value** | **95.0% Confidence Interval for B** | |
| --- | --- | --- | --- | --- |
|  |  |  | **Lower Bound** | **Upper Bound** |
| Educational level (secondary vs. intermediate or lower) | 0.284 | **0.024** | 0.038 | 0.531 |
| Educational level (university/postgraduate vs. intermediate or lower) | 0.190 | 0.086 | -0.027 | 0.407 |
| Place of residence (Mount Lebanon vs. Beirut) | -0.115 | 0.384 | -0.373 | 0.143 |
| Place of residence (North/Akkar vs. Beirut) | -0.288 | 0.087 | -0.617 | 0.042 |
| Place of residence (South/Nabatiyeh vs. Beirut) | -0.023 | 0.843 | -0.251 | 0.205 |
| Place of residence (Baalbek/Beqaa vs. Beirut) | -0.031 | 0.858 | -0.369 | 0.307 |
| Being a healthcare professional | 0.169 | 0.163 | -0.068 | 0.407 |
| Monthly household income (250 to 500 vs. < 250 USD) | 0.205 | 0.064 | -0.012 | 0.422 |
| Monthly household income (500 to 1000 vs. < 250 USD) | -0.003 | 0.983 | -0.276 | 0.270 |
| Monthly household income (> 1000 vs. < 250 USD) | 0.032 | 0.853 | -0.307 | 0.371 |
| Social support (moderate vs. low) | 0.318 | 0.051 | -0.002 | 0.638 |
| Social support (high vs. low) | 0.881 | **<0.001** | 0.549 | 1.213 |
| Psychological distress | -0.017 | **0.036** | -0.033 | -0.001 |

A P-value of less than 0.05 was considered significant.
