# Supplementary material for: Coping strategies among family caregivers of community-dwelling older adults in Lebanon amid the economic crisis
Source: PLoS One. 2026 Jan 23;21(1):e0340972. doi: 10.1371/journal.pone.0340972 (PMC12829931; doi:10.1371/journal.pone.0340972)
Supplement: S6 Table — (DOCX) [file pone.0340972.s006.docx]

**S6 Table.** Factors associated with avoidance coping strategies among caregivers of community-dwelling older adults

|  | **Unstandardized Coefficients B** | **P-value** | **95.0% Confidence Interval for B** | |
| --- | --- | --- | --- | --- |
|  |  |  | **Lower Bound** | **Upper Bound** |
| Caregivers’ age | 0.008 | 0.082 | -0.001 | 0.016 |
| Caregivers’ gender | 0.200 | 0.076 | -0.021 | 0.421 |
| Place of residence (Mount Lebanon vs. Beirut) | -0.121 | 0.286 | -0.344 | 0.102 |
| Place of residence (North/Akkar vs. Beirut) | -0.308 | **0.031** | -0.588 | -0.027 |
| Place of residence (South/Nabatiyeh vs. Beirut) | 0.020 | 0.843 | -0.177 | 0.217 |
| Place of residence (Baalbek/Beqaa vs. Beirut) | -0.142 | 0.382 | -0.428 | 0.143 |
| Working status | -0.008 | 0.921 | -0.164 | 0.148 |
| Caregiver’s relationship with the care recipient (grandchild vs. son/daughter) | -0.050 | 0.720 | -0.321 | 0.221 |
| Caregiver’s relationship with the care recipient (son/daughter-in-law vs. son/daughter) | 0.119 | 0.259 | -0.087 | 0.325 |
| Caregiver’s relationship with the care recipient (spouse vs. son/daughter) | 0.411 | 0.137 | -0.131 | 0.953 |
| Caregiver’s relationship with the care recipient (others vs. son/daughter) | 0.305 | 0.079 | -0.036 | 0.646 |
| Daily caregiving time (hour) | -0.007 | 0.237 | -0.017 | 0.004 |
| Psychological distress | 0.065 | **<0.001** | 0.051 | 0.079 |
| Number of chronic diseases | 0.014 | 0.459 | -0.023 | 0.051 |
| ADL score | -0.013 | 0.553 | -0.057 | 0.031 |

A P-value of less than 0.05 was considered significant
